# Unfractionated Heparin Promotes Osteoclast Formation *in Vitro* by Inhibiting Osteoprotegerin Activity

**DOI:** 10.3390/ijms17040613

**Published:** 2016-04-22

**Authors:** Binghan Li, Dan Lu, Yuqing Chen, Minghui Zhao, Li Zuo

**Affiliations:** 1 Renal Division, Peking University First Hospital, No. 8 Xishiku Street, Xi Cheng District, Beijing 100034, China; libinghanice@bjmu.edu.cn (B.L.); chenyuqing112@hotmail.com (Y.C.); mhzhao@bjmu.edu.cn (M.Z.); 2 Peking University Institute of Nephrology, Beijing 100034, China; 3 Key Laboratory of Renal Disease, Ministry of Health, Beijing 100034, China; 4 Key Laboratory of Chronic Kidney Disease Prevention and Treatment, Peking University, Ministry of Education, Beijing 100034, China; 5 Department of Cardiology, Peking University First Hospital, No. 8 Xishiku Street, Xi Cheng District, Beijing 100034, China; ludan39@foxmail.com

**Keywords:** unfractionated heparin, osteocyte, osteoclast, osteoclastogenesis, osteoprotegerin (OPG)

## Abstract

Heparin has been proven to enhance bone resorption and induce bone loss. Since osteoclasts play a pivotal role in bone resorption, the effect of heparin on osteoclastogenesis needs to be clarified. Since osteocytes are the key modulator during osteoclastogenesis, we evaluated heparin’s effect on osteoclastogenesis *in vitro* by co-culturing an osteocyte cell line (MLO-Y4) and pre-osteoclasts (RAW264.7). In this co-culture system, heparin enhanced osteoclastogenesis and osteoclastic bone resorption while having no influence on the production of RANKL (receptor activator of NFκB ligand), M-CSF (macrophage colony-stimulating factor), and OPG (osteoprotegerin), which are three main regulatory factors derived from osteocytes. According to previous studies, heparin could bind specifically to OPG and inhibit its activity, so we hypothesized that this might be a possible mechanism of heparin activity. To test this hypothesis, osteoclastogenesis was induced using recombinant RANKL or MLO-Y4 supernatant. We found that heparin has no effect on RANKL-induced osteoclastogenesis (contains no OPG). However, after incubation with OPG, the capacity of MLO-Y4 supernatant for supporting osteoclast formation was increased. This effect disappeared after OPG was neutralized and reappeared after OPG was replenished. These results strongly suggest that heparin promotes osteocyte-modulated osteoclastogenesis *in vitro*, at least partially, through inhibiting OPG activity.

## 1. Introduction

Heparin, a highly-sulfated glycosaminoglycan, is an anticoagulant that is widely used in various thrombotic diseases and during hemodialysis [1]. Its administration can induce bone volume loss and decreased bone density [2]. This adverse effect is critical for patients undergoing long-term heparin therapy, such as maintenance hemodialysis patients.

Our previous study of rats with chronic kidney disease (CKD) confirmed that heparin treatment could induce bone loss, and further suggested that increased osteoclastic bone resorption may be involved in this effect [3]. Therefore, we aimed to investigate the effects of heparin on osteoclast formation and activity *in vitro*.

Osteoclasts differentiate from the hematopoietic precursors of the monocyte-macrophage lineage [4]. Receptor activator of NFκB ligand (RANKL; also called TRANCE, ODF, and OPGL), osteoprotegerin (OPG), and macrophage colony-stimulating factor (M-CSF) are the most important factors regulating osteoclast formation [4]. RANKL is essential for osteoclast formation and survival [5]. Since RANKL is produced by osteoblasts and osteocytes, both could support osteoclast formation [6,7,8].

Compared with osteoblasts, osteocytes have a much more complex physiological function [9,10,11,12,13,14], which suggests that osteocytes are not simply the end-stage or dormant state of osteoblasts but a newly recognized critical modulator of bone metabolism. More importantly, in recent years, many studies have discovered that osteocytes, rather than osteoblasts, play the key role in osteoclast formation [15,16]. Contrary to the previous understanding, RANKL produced by osteoblasts or their progenitors does not contribute to adult bone remodeling, while osteocytes are essential sources of RANKL, which controls osteoclast formation [15]. In fact, osteocytes have been found to act as an orchestrator of bone remodeling through the regulation of both osteoclast and osteoblast activity [11]. Unfortunately, previous investigations mostly concentrated on osteoblasts and their interaction with heparin, while studies that focus on the influence of heparin on osteocyte-modulated osteoclastogenesis are as rare as hen’s teeth. Thus, although Atsushi Irie and colleague’s investigation of the effects of heparin on osteoblast-induced osteoclastogenesis in 2007 is convincing [17], osteocytes and their osteoclastogenic modulatory function under the influence of heparin remains unclear and requires further study.

Many proteins can bind with heparin *in vivo*. Previous studies have confirmed that OPG is one such heparin-binding protein [17,18]. In 2006, Theoleyre proved that OPG can bind to heparin with a high affinity (KD: 0.28 nM) by using surface plasmon resonance technology [18]. In 2007, by applying heparin-sepharose, Atsushi further confirmed that OPG could specifically bind to heparin [17]. More importantly, the researchers discovered that the binding of heparin and OPG interfered with osteoclastogenesis in a co-culture of osteoblasts and pre-osteoclasts [17]. Although the newest opinion is that osteoblasts are not responsible for regulating the differentiation of osteoclasts *in vivo*, Atsushi’s finding still has merit. Their results inspires us to ask whether heparin influences osteocytes’ regulation of osteoclastogenesis by a similar mechanism (binding with OPG), or by some other new pathway.

Since osteocytes are embedded in bone mineral matrix, immortal osteocyte-like cell lines are widely used. An MLO-Y4 mouse osteocyte-like cell line was established by Bonewald and colleague in 1997 [19]. This cell line can support osteoclast formation *in vitro*, by secreting RANKL, OPG, and M-CSF, without supplementation from any other regulatory factors [20]. The RAW 264.7 monocyte cell line is the most commonly used osteoclast precursor; it is widely used to examine osteoclast formation *in vitro* [21,22,23,24,25].

In the present study, we performed a co-culture of osteocytes (MLO-Y4) and pre-osteoclasts (RAW264.7) and added various concentrations of heparin to investigate the effects of heparin on osteoclast formation and activation. We then further explored the mechanism related to these effects.

## 2. Results

### 2.1. Heparin Enhances Osteoclastogenesis and Osteoclastic Bone Resorption

**Figure 1:**
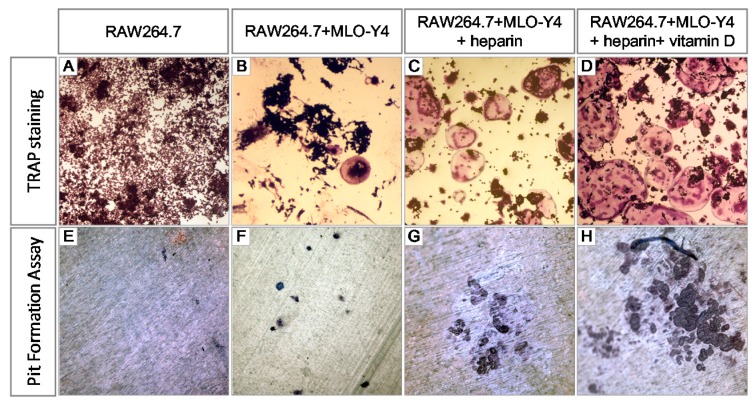
Heparin promotes osteocyte-induced osteoclast formation. (**A**) RAW264.7 were cultured alone, in the absence of both MLO-Y4 cells and RANKL; Eight days later (**B**–**D**) RAW 264.7 were co-cultured with MLO-Y4 for eight days, with or without the addition of 10 IU/mL heparin and 10 nM 1,25(OH)_2_D_3_. The osteoclasts were stained for TRAP (80×); and (**E**,**H**) RAW264.7 were cultured with or without MLO-Y4 on dentin slice, in the presence or absence of 10 IU/mL heparin and 10 nM 1,25(OH)_2_D_3_. The resorption pits were stained with toluidine blue (200×).TRAP: tartrate resistant acid phosphatase.

MLO-Y4 osteocyte-like cells could support osteoclast formation and activation in the absence of any other exogenous osteotropic factors [20]. To test the effects of heparin on osteocyte-modulated osteoclastogenesis, we co-cultured MLO-Y4 cells and RAW264.7 cells in the absence of any exogenous regulatory factors with the addition of various concentrations of heparin. The osteoclasts were defined as tartrate-resistant acid phosphatase (TRAP) positive cells with more than three nuclei. As Figure 1A,E show (both without MLO-Y4 cells), when RAW264.7 cells were monocultured in the absence of any RANKL, neither osteoclasts nor resorption pits were observed. While in a co-culture of MLO-Y4 and RAW264.7, osteoclasts were generated (deep red cell in Figure 1B) and osteoclastic resorption pits were formed on the dentin slice (deep blue in Figure 1F). In the co-culture system, heparin treatment resulted in increased osteoclast formation (Figure 1C), and more osteoclastic bone resorption pits were formed on the dentin slices (Figure 1G).

The capacity for promoting osteoclastogenesis with MLO-Y4 could be enhanced with the addition of 1,25(OH)_2_D_3_ [20]. Although the exact mechanism behind this phenomenon is not well understood, it is clear that M-CSF production increases in MLO-Y4 after treatment with 1,25(OH)_2_D_3_ [20]. Moreover, 1,25(OH)_2_D_3_ is known to regulate RANKL expression in stromal cells and osteoblast [26,27,28], so it may serve the same role in osteocytes.

Although in the 1,25(OH)_2_D_3_-free co-culture of MLO-Y4 and RAW264.7, heparin’s effect on osteoclastogenesis was tested without any interference, we also observed heparin’s effect on osteoclastogenesis in the presence of 1,25(OH)_2_D_3_ for the two conditions. First, with the addition of 1,25(OH)_2_D_3_, the capacity of MLO-Y4 to support osteoclastogenesis is amplified; therefore, the results will be observed more easily. Second, because 1,25(OH)_2_D_3_ can be synthesized locally in bone tissue, we assume that adding active vitamin D will make our co-culture system more closely approximate the environment of osteoclast formation. As Figure 1D,H indicate, with the addition of 1,25-dihydroxyvitamin D_3_ (1,25(OH)_2_D_3_), the osteoclasts became larger and more numerous, and more resorption pits were formed on the dentin slices.

**Figure 2:**
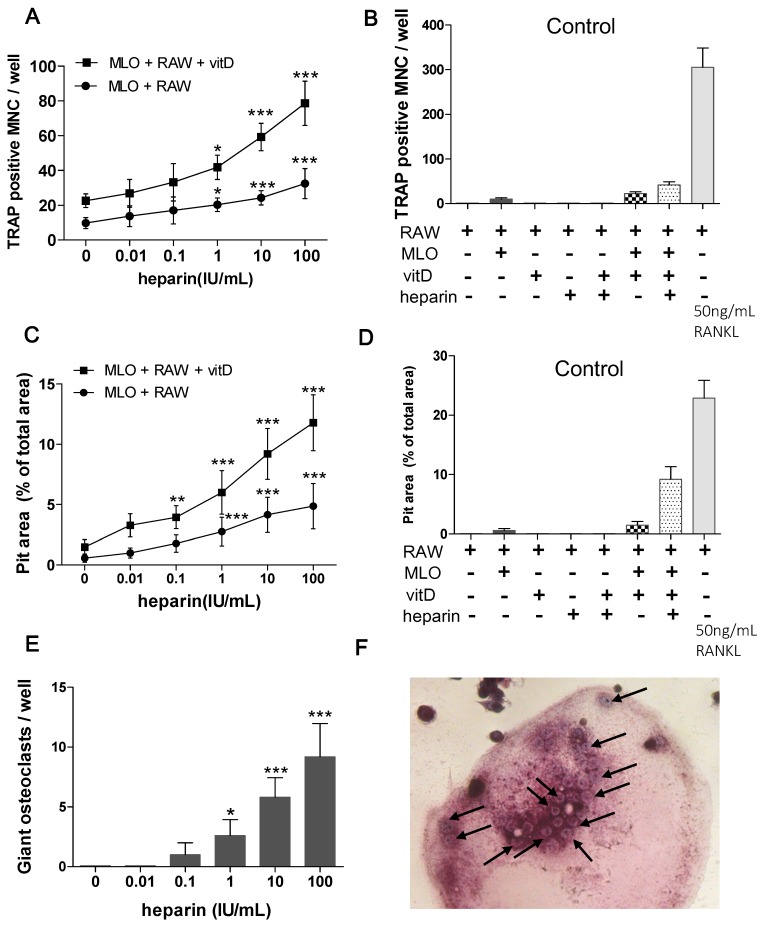
Quantitative analysis of osteoclast formation and the pit formation assay in experiment groups and control groups. In experiment groups, MLO-Y4 and RAW264.7 were co-cultured on a plastic dish or dentin slice for eight days, treated with different level of heparin, in the absence or presence of 10 nM 1,25(OH)_2_D_3_. In control groups, RAW264.7 were cultured with or without MLO-Y4, in the absence or presence of 10 IU/mL heparin and 10 nM 1,25(OH)_2_D_3_. 50 ng/mL RANKL induced osteoclastogenesis served as a positive control. (**A**) Osteoclast numbers per well in experiment groups (*n* = 7); (**B**) osteoclast numbers per well in control groups (*n* = 5); (**C**) area of osteoclastic resorption pits on dentin slices in experiment groups (*n* = 3); (**D**) area of osteoclastic resorption pits on dentin slices in control groups (*n* = 3); (**E**) giant osteoclasts (osteoclast with more than 20 nuclei) number in experiment group supplemented with 1,25(OH)_2_D_3_ (*n* = 5); and (**F**) giant osteoclast (200×). The black arrows indicate the nuclei in a single giant osteoclast. Each of above values is expressed as the mean ±S.D. MLO: MLO-Y4 cells. RAW: RAW264.7 cells. vitD: 1,25(OH)_2_D_3_. TRAP: tartrate resistant acid phosphatase. MNC: multinuclear cell. * *p* < 0.05; ** *p* < 0.01; *** *p* < 0.001 represent significant differences from the relevant group 0.

Further quantitative analysis revealed that as the heparin concentration increased, more osteoclasts were formed (Figure 2A) and more resorption pits were also observed (Figure 2C). To eliminate the effect of interference factor, several control groups were established. In control groups, RAW264.7 cells were cultured with or without MLO-Y4, in the absence or presence of 10 IU/mL heparin or 10 nM 1,25(OH)_2_D_3_ (Figure 2B,D). 50 ng/mL RANKL induced osteoclastogenesis served as the positive control. The results for the control groups suggested that without the support of MLO-Y4 cells, RAW264.7 cells could not differentiate into osteoclasts (Figure 2B, column 1 and column 3–5). Neither heparin nor 1,25(OH)_2_D_3_ could induce RAW264.7 cells to form osteoclasts or resorption pits (Figure 2B,D).

Moreover, in groups supplemented with 1,25(OH)_2_D_3_, the diameter of the osteoclasts was significantly larger. To assess the effect of heparin on the size of the generated osteoclasts, the number of giant osteoclasts (osteoclasts with more than 20 nuclei) was also counted. In the groups supplemented with 1,25(OH)_2_D_3_, as the heparin concentration increased, more giant osteoclasts were formed (Figure 2E,F).

### 2.2. Heparin Has No Effect on the Production of RANKL, OPG, and M-CSF

**Figure 3:**
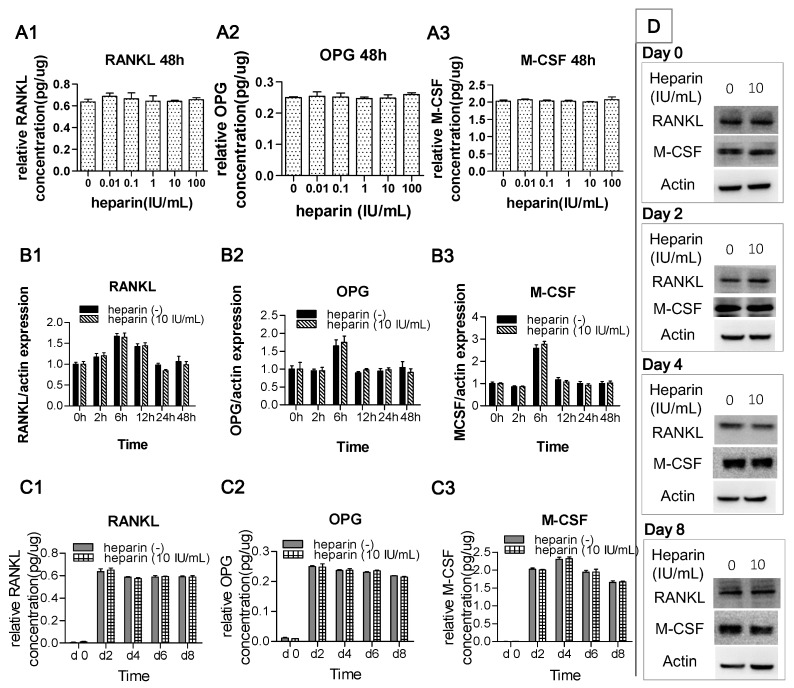
Heparin has no effect on the production of RANKL, OPG, and M-CSF. (**A1**–**A3**) The concentration of factors in the supernatant of the co-culture system, determined with ELISA at 48 h. The medium concentrations of RANKL, OPG, and M-CSF in group 0 were 416, 164, and 1327 pg/mL. After normalized by total proteins, no significant difference among each group (*p* > 0.05); (**B1**–**B3**) The relative expression of RANKL, OPG, and M-CSF mRNA in co-culture cells. The values are expressed as relative expression levels compared with the 0 h groups. At each time point, no significant difference between group 0 and group 10 (*p* > 0.05); (**C1**–**C3**) concentration of the factors in the supernatant of the co-culture system at various time point, determined with ELISA. At each time point, no significant difference between group 0 and group 10 (*p* > 0.05); and (**D**) MLO-Y4 and RAW 264.7 were co-cultured with or without 10 IU/mL heparin for eight days. The protein expression of RANKL and M-CSF were analyzed by Western blot at day 0, 2, 4, and 8. Relative band intensity of RANKL and OPG to β-actin were quantified and no significant difference between group 0 and group 10 at each time point (*n* = 3; *p* > 0.05). Each of the above values is expressed as the mean ± S.D. RANKL: receptor activator of NFκB ligand; OPG: osteoprotegerin; M-CSF, macrophage colony-stimulating factor.

Since osteocytes modulate osteoclastogenesis through the expression of RANKL, M-CSF, and OPG, we further detected the influence of heparin treatment on their production. Without heparin, the concentrations of RANKL, M-CSF, and OPG in co-culture supernatants on 48 h were 416, 164, and 1327 pg/mL. In the first 48 h, after adjustment for total proteins of cells, the concentrations of RANKL, M-CSF, and OPG in co-culture supernatants were not affected by heparin treatment (Figure 3A1,A3) and the relative mRNA expression of these factors at 0, 2, 6, 12, 24, and 48 h also showed no difference between the co-culture groups treated with or without heparin (Figure 3B1,B3).

In the co-cultures, the level of RANKL, M-CSF, and OPG were assessed on day 2, 4, 6, and 8 in both the culture supernatants and cell lysates. At each time point, no significant differences between the 0 IU/mL heparin group and the 10 IU/mL heparin group were observed (Figure 3C1,C3,D).

### 2.3. Heparin Has No Effects on RANKL Induced Osteoclast Formation

**Figure 4:**
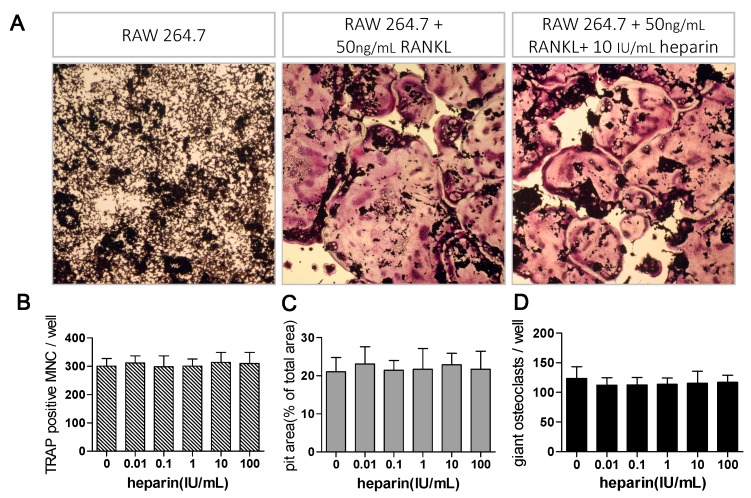
Heparin has no effect on recombinant RANKL-induced osteoclast formation. (**A**) RAW264.7 cells were monocultured for six days, with or without 50 ng/mL recombinant RANKL and 10 IU/mL heparin. The osteoclasts were stained for TRAP (80×); (**B**–**D**) RAW 264.7 cells were monocultured under stimulation of 50 ng/mL recombinant RANKL, with addition of different level of heparin. Osteoclast number, pit area, and giant osteoclast number were quantified in each group. No significantly different among each group (*n* = 5). MNC: multinuclear cell; TRAP: tartrate-resistant acid phosphatase; RANKL: receptor activator of NFκB ligand; Each of the above values is expressed as the mean ± S.D.

To test whether heparin directly affect osteoclast formation, RAW264.7 cells were monocultured and induced by recombinant RANKL. Since RAW264.7 cells were cultured without the presence of MLO-Y4 cells, osteoclastogenesis was induced in the absence of any OPG. As shown in Figure 4A, 50 ng/mL recombinant RANKL effectively induced osteoclast formation, while osteoclasts formation were not altered by heparin treatment. Moreover, the quantitative analysis of pit area and giant osteoclast also suggested that heparin has no effects on pit resorption activity and giant osteoclast number (Figure 4B).

### 2.4. The Effect of Heparin Was Related to the Inhibition of OPG Activity

As the above results indicate, heparin promotes MLO-Y4 cell-modulated osteoclast formation but has no effects on RANKL-induced osteoclastogenesis or regulatory factor production. According to other studies, heparin has the potential to bind and interact with a host of proteins [29]. In fact, OPG is a “heparin-binding protein”, and interaction with heparin may alter the protein’s distribution and function [17,18,30].

Based on these findings, we hypothesized that a similar heparin-induced blockage of OPG activity also occurs in the co-culture system and that heparin would have a promotional effect on MLO-Y4-modulated osteoclast formation as a result. To test this hypothesis, we performed several additional tests, with the following results.

#### 2.4.1. The “Saturation Phenomenon”

**Figure 5:**
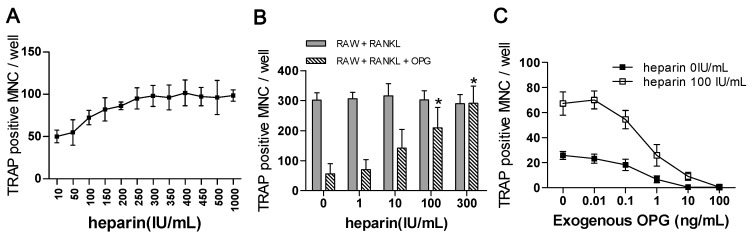
Characteristics of heparin’s effect on osteoclast formation. (**A**) Osteoclast numbers in a co-culture of RAW264.7 and MLO-Y4, treated with higher level of heparin. When heparin concentration is over 250 IU/mL, no significance among each group (*n* = 3, *p* = 0.993); (**B**) RAW264.7 were monocultured and induced with 50 ng/mL RANKL, with or without replenishment of 10 ng/mL exogenous OPG. The effects of heparin treatment on osteoclast formation were quantified (*n* = 3); (**C**) The number of osteoclasts in co-cultured system supplemented with various concentration of OPG. MNC: multinuclear cell; TRAP: tartrate-resistant acid phosphatase; RANKL: receptor activator of NFκB ligand; Each of the above values is expressed as the mean ± S.D. * *p* < 0.05 represent significant differences from the relevant group 0.

In the co-cultures, with increased heparin concentrations, the number of osteoclasts remained stable. No significant differences were observed among the groups treated with more than 250 IU/mL heparin (*n* = 3, *p* = 0.993; Figure 5A), which suggests that the heparin effect is subject to a saturation phenomenon.

#### 2.4.2. The Effects of Heparin on RANKL-Induced Osteoclastogenesis with the Addition of OPG

As mentioned before, in the RAW264.7 monoculture system, which contains no OPG, heparin has no effect on osteoclast formation (Section 2.3). In contrast, when OPG is replenished in monoculture medium, heparin had a promotional effect on osteoclastogenesis (Figure 5B, diagonally striped columns). The osteoclast counts of the 100 and 300 IU/mL groups differed significantly from those of the 0 and 1 IU/mL groups (*p* < 0.01). These results suggest the OPG might be the target of heparin.

#### 2.4.3. The Use of Exogenous OPG to Attenuate the Effects of Heparin

With the replenishment of OPG in the co-culture, the number of osteoclasts gradually decreased in the groups that were (solid square) or were not (hollow square) treated with heparin (Figure 5C). Moreover, with increased OPG concentrations, the number of formed osteoclasts in the heparin-treated groups gradually decreased, suggesting that the addition of exogenous OPG to co-cultures could attenuate the effects of heparin.

#### 2.4.4. Osteoclast Formation Induced by the Conditioned Media of MLO-Y4 Cells

The function of osteocytes appears to be intricate. Despite producing classical regulatory proteins, namely RANKL, M-CSF, and OPG, osteocytes may also modulate osteoclastogenesis by secreting some unknown regulatory factors. Hence, heparin could act on those unknown controlling factors in a way that interferes with their production while leaving the production of RANKL, M-CSF, and OPG alone.

**Figure 6:**
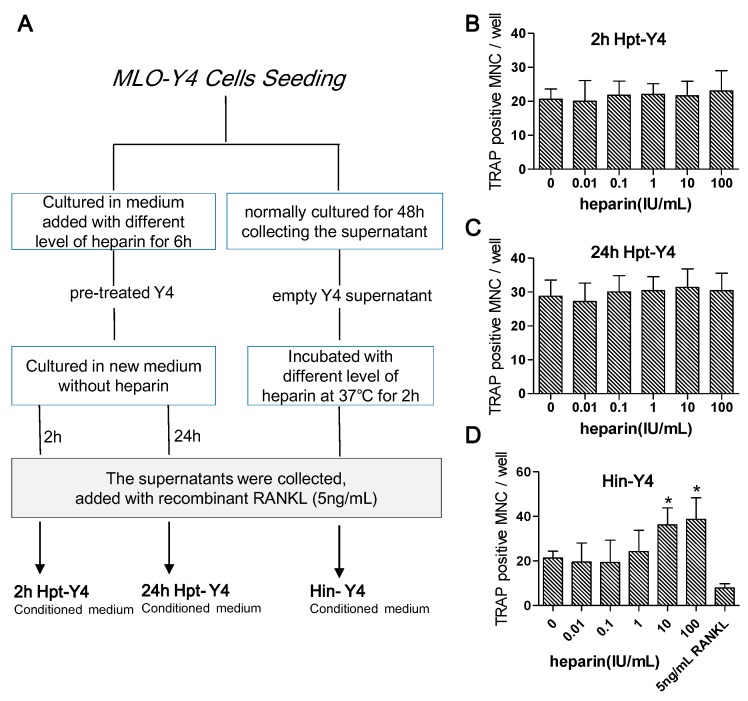
The effects of heparin on osteoclastogenesis induced by MLO-Y4 conditioned medium. (**A**) MLO-Y4 conditioned medium preparation flowchart. MLO-Y4 cells were pre-treated with heparin for 6 h, then cultured in fresh medium. The supernatant were then collected after 2 h (2h Hpt-Y4) and 24 h (24h Hpt-Y4). The preparation process of Hin-Y4 conditioned medium were displayed on the right flow line. MLO-Y4 cells were cultured for 48 h and the supernatant were collected as “empty Y4 supernatant”. Empty Y4 supernatant was then incubated with various concentrations of heparin to obtain heparin-incubated MLO-Y4-conditioned medium (Hin-Y4). Each conditioned medium was added with recombinant RANKL to make sure the osteoclast can form; (**B**,**C**) RAW264.7 cells were cultured in 2h Hpt-Y4 and 24h Hpt-Y4, and the osteoclast number were counted eight days later. (*n* = 3). No significance among each groups (*p* > 0.05); (**D**) RAW 264.7 were cultured in Hin-Y4 for eight days, and osteoclasts in each group were counted. (*n* = 3). osteoclastogenesis induced by 5 ng/mL recombinant RANKL was served as a control. MNC: multinuclear cell; TRAP: tartrate-resistant acid phosphatase; RANKL: receptor activator of NFκB ligand; OPG: osteoprotegerin; each of the above values is expressed as the mean ± S.D. * *p* < 0.05 represent significant differences from the relevant group 0.

We conducted an experiment to examine this possibility. In this experiment, we first pre-treated the MLO-Y4 cells with gradient levels of heparin. Then cultured those pre-treated MLO-Y4 cells in fresh medium. Later, we collected the supernatant after two and 24 h to create two types of heparin pre-treated conditioned medium (2h Hpt-Y4 and 24h Hpt-Y4; see details in Figure 6A). We cultured RAW 264.7 for eight days in those two types of medium, and finally observed the formation of osteoclasts. However, the number of osteoclasts remained the same with every heparin level, as shown in Figure 6B,C, suggesting heparin has no influence on the production of unknown regulatory factors.

MLO-Y4 could secret many known (such as RANKL, M-CSF and OPG) and unknown regulatory factors into the supernatant. As a consequence, this type of supernatant could induce RAW264.7 to differentiate into osteoclasts. To investigate the role of heparin in this differentiation, we prepared heparin incubated MLO-Y4 conditioned medium (Hin-Y4; details in Figure 6A). Briefly, we first collected the supernatant of MLO-Y4, which was normally cultured for 48 h. We then harvested the Hin-Y4 by incubating the supernatant with various concentrations of heparin. Lastly, we cultured RAW264.7 in Hin-Y4 for eight days and observed the number of newly formed osteoclasts using TRAP staining. We discovered that the increase in the number of osteoclasts paralleled the increase of the heparin gradient level, which suggests that heparin had a promotional effect on MLO-Y4 supernatant-induced osteoclastogenesis (Figure 6D).

**Figure 7:**
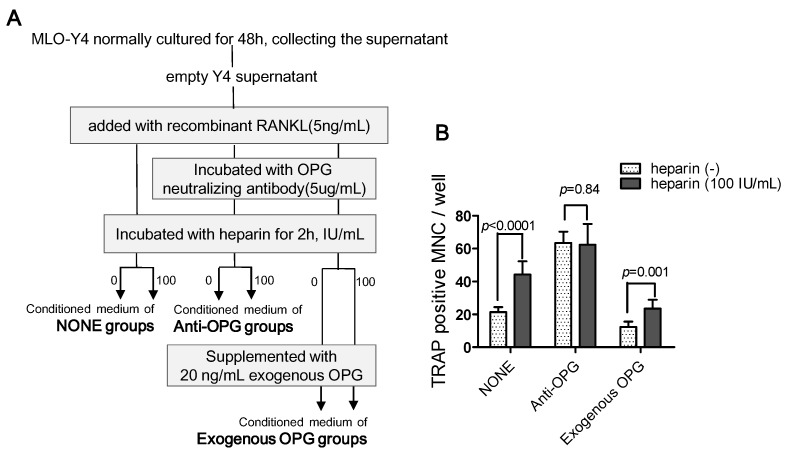
OPG is essential in the ability of heparin to enhance osteoclast formation. (**A**) Empty Y4 supernatant were incubated with or without OPG neutralizing antibody, heparin, and exogenous OPG to obtain conditioned medium used in NONE, anti-OPG, and exogenous OPG groups. Each conditioned medium were added with recombinant RANKL to make sure the osteoclast can formed; (**B**) RAW264.7 were cultured in three types of conditioned medium and osteoclast formation was assessed (*n* = 3). MNC: multinuclear cell; TRAP: tartrate-resistant acid phosphatase; RANKL: receptor activator of NFκB ligand; OPG: osteoprotegerin; Each of the above values is expressed as the mean ± S.D.

The different heparin effects in Hpt-Y4 and Hin-Y4 suggest that heparin changes the activity of certain regulatory factors that affect osteoclastogenesis in downstream. As described previously, many studies have confirmed that OPG is a heparin binding protein, which implies that OPG might play a pivotal role in heparin’s influence. To determine whether OPG was essential to heparin’s effect, we added OPG-neutralizing antibody to the supernatant of MLO-Y4 and then re-assessed the influence of heparin on osteoclast formation induced by the MLO-Y4 supernatant (Figure 7A). As Figure 7B shows, in the NONE groups, heparin enhanced osteoclast formation (*p* < 0.0001), while in the anti-OPG groups, after OPG was neutralized with a specific antibody, the number of osteoclasts was no longer influenced by heparin treatment (Figure 7B, anti-OPG columns, *p* = 0.8471). Moreover, the replenishment of exogenous OPG resulted in the reappearance of heparin effect (Figure 7B, Exogenous OPG columns, *p* = 0.001). These results strongly suggest that OPG is essential to the ability of heparin to enhance osteoclast formation.

In summary, the results of these experiments strongly support our hypothesis that the inhibition of OPG activity is a possible mechanism of heparin’s effects.

## 3. Discussion

Our results demonstrate that heparin promotes MLO-Y4-induced osteoclast formation without affecting the production of classical regulatory factors. The effects of heparin showed a “saturation phenomenon”.

This effect disappeared when OPG was neutralized and reappeared with exogenous OPG supplementation, which strongly suggests the involvement of OPG. Interestingly, the supernatant of heparin pre-treated MLO-Y4 cells showed no increase in the ability to induce osteoclast formation, thus excluding the possibility that heparin could alter the production of unknown soluble factors. Thus, we can conclude that the inhibition of OPG activity is, at least in part, a possible mechanism of heparin’s ability to promote MLO-Y4-induced osteoclast formation.

Although our results do not support the assumption that heparin could alter the production of OPG and RANKL, in clinical studies of hemodialysis patients [31,32] and healthy students [30], plasma levels of OPG and soluble RANKL were influenced by treatment with both heparin and low molecular weight heparin. There are two possible explanations for these inconsistent results. First, heparin may alter the concentrations of RANKL and OPG in serum by changing the distribution of OPG and RANKL rather than by changing their production. Second, many cells other than osteocytes can synthesize OPG and RANKL. Therefore, heparin might exert some effect on production of OPG and RANKL by other cells, resulting in the plasma level changes in OPG and RANKL.

Osteoclast formation is modulated by both osteocytes and osteoblasts, and osteocytes are now believed to play the key role in the process of osteoclast differentiation *in vivo* [15]. However, in past decades, because osteocyte function was difficult to study, much attention focused on the role of osteoblasts rather than osteocytes in osteoclast differentiation. In 2007, Atsushi and colleagues studied the effects of heparin on osteoblast-induced osteoclast formation [17]. Similar to our pit formation assay results, they found that heparin could enhance the bone resorption activity of osteoclasts induced by osteoblasts. However, in their study, TRAP activity was not affected by heparin treatment; therefore, they concluded that heparin enhances osteoblast-induced osteoclastic activity but not osteoclastogenesis. On the contrary, our study showed that the number of generated osteoclasts increased with the addition of heparin. This result indicates that heparin has the potential to promote osteocyte-induced osteoclast formation. One possible explanation for this difference between the two studies is that osteoblasts and MLO-Y4 cells have different patterns of OPG production. In the study of Atsushi, the real-time PCR results show that OPG expression gradually increased and reached a maximum on day 5, at which time the majority of the cells have already differentiated [17]. In comparison, our ELISA data for OPG showed that maximum production was reached on day 2 and then slightly and slowly decreased (Figure 3C2), which would allow heparin to inhibit OPG production from the very beginning.

Osteocytes regulate osteoclast formation not only through RANKL/OPG interaction but also through osteocyte apoptosis. Apoptotic osteocytes can modulate the differentiation and recruitment of osteoclast precursors [33]. Such activity is involved in the pathophysiological process of osteoporosis caused by various factors [34,35,36]. However, our results imply that modulation via osteocyte apoptosis is not the mechanism underlying the promotional effects of heparin. Osteocyte death is usually accompanied by increased RANKL production [37], but in our study, heparin administration did not affect the production of RANKL (Figure 3).

The purpose of our study was to investigate the effect of heparin on osteocyte-modulated osteoclastogenesis. Our experiments had several limitations: (1) we performed a co-cultured system of an immortalized osteocyte-like cell line and a monocytic cell line to investigate the *in vitro* effects of heparin; however, *in vitro* experiments cannot completely simulate the *in vivo* environment; (2) Our study indicated that heparin has no influence on the production of traditional regulatory factors (RANKL, OPG, and M-CSF), whether in soluble form or in membrane-bound form. Nevertheless, some unknown regulatory factors produced by osteocytes are very likely to exist and exert certain modulatory functions in the osteoclastogenesis process. In our study, the results of verification test 4 excluded the possibility that heparin alters the production of unknown soluble factors, while the possible effects of heparin on unknown factors binding with the osteocyte membrane could not be verified. This possible mechanism requires further investigation.

In summary, we co-cultured osteocyte-like MLO-Y4 cells and osteoclast precursors to study the effects of heparin on osteocyte-induced osteoclast formation. Our findings suggest that heparin promotes MLO-Y4 cell-induced osteoclast formation and bone resorption activity. Heparin had no influence on the production of RANKL, OPG and M-CSF; however, heparin treatment increased the osteoclastogenesis induced by the MLO-Y4 supernatant, and these effects disappeared when OPG was neutralized with anti-OPG agents. These findings support our hypothesis that heparin inhibits OPG activity through binding and, thus, enhances osteoclast differentiation.

## 4. Materials and Methods

### 4.1. Materials

The cell culture media, rat tail collagen type I, was purchased from Gibco BRL (Grand Island, NY, USA); FBS and calf serum (CS) were purchased from Hyclone Laboratories, Inc. (Logan, UT, USA). Anti-RANKL, anti-actin antibodies, HRP-conjugated secondary antibodies, the ELISA kit for mouse RANKL, OPG, and M-CSF were purchased from Abcam (Cambridge, UK). Anti-OPG, anti-M-CSF, recombinant RANKL, and recombinant M-CSF were purchased from R and D Systems (Minneapolis, MN, USA). Unfractionated heparin for clinical use (heparin sodium injection, 2 mL: 12,500 IU), which is derived from porcine mucosa, and 1,25-dihydroxyvitamin D3 (1,25(OH)_2_D_3_) were purchased from Sigma-Aldrich (St. Louis, MO, USA). Dentin slices were purchased from Immunodiagnostic Systems Limited (Boldon, UK).

### 4.2. Cell Culture

With the approval of Bonewald (University of Missouri-Kansas City), the MLO-Y4 cell line was delivered by Peng Shang (School of Life Sciences, Northwestern Polytechnical University, Xi’an, China). The MLO-Y4 cells were cultured in α-MEM medium supplemented with 2.5% FBS and 2.5% calf serum, including 1% penicillin-streptomycin. The RAW264.7 cells were purchased from the Cell Bank of the Chinese Academy of Medical Sciences and cultured in DMEM medium supplemented with 10% FBS and 1% penicillin-streptomycin.

### 4.3. Co-Culture of MLO-Y4 Cells with RAW 264.7 Cells

MLO-Y4 cells (1000–1500 cells/cm^2^) were seeded in 48-well plates two days before the addition of RAW264.7 cells. RAW264.7 cells were seeded at 2000–2500 cells/cm^2^ (this day was marked as day 0). These cells were co-cultured in alpha-MEM medium plus 10% FBS, supplemented with or without 10 nM of 1,25(OH)_2_D_3_ for eight days. The culture medium was replaced every two days. To test the effects of heparin treatment, various concentrations of heparin were added to the co-cultured medium. Co-culturing was terminated on day 8, and the cells were fixed and stained with tartrate-resistant acid phosphatase (TRAP).

To eliminate the effect of interference factor, several control groups were established. RAW264.7 cells were cultured alone or co-cultured with MLO-Y4 cells, in the presence or absence of 10 nM 1,25(OH)_2_D_3_ or 10 IU/mL heparin. RAW264.7 cells cultured in medium with 50 ng/mL recombinant RANKL served as the positive control.

In some other experiments, the RAW264.7 cells were cultured alone and induced with 50 ng/mL soluble recombinant RANKL for six days. The effects of different levels of heparin on RANKL-induced osteoclast formation were also determined.

### 4.4. Tartrate-Resistant Acid Phosphate (TRAP) Stain Assay

When the co-culturing or monoculturing was terminated, the cells were fixed and stained for TRAP using a phosphatase leukocyte kit (Sigma-Aldrich). The number of osteoclasts (TRAP-positive cells with more than three nuclei) and giant osteoclasts (osteoclasts with more than 20 nuclei) in every well was counted.

### 4.5. Pit Formation Assay

As described above, MLO-Y4 cells (1000–1500 cells/cm^2^) and RAW264.7 cells (2000–2500 cells/cm^2^) were co-cultured on dentin slices (5 mm in diameter, 0.3 mm in thickness) in 96-well plates for three days, with or without the addition of 10 nM 1,25(OH)_2_D_3_ and different levels of heparin. Then the dentin slices were transferred into 48-well plates and cultured for five more days. In some experiments, RAW264.7 cells were monocultured on dentine slices in 96-well plates for six days and treated with 50 ng/mL recombinant RANKL and various concentration of heparin. The cells were then gently removed with a writing brush, and the dentin slices were fixed with 4% glutaraldehyde and stained with 1% toluidine blue. Each slice was observed under reflected-light microscope and photographed (four quadrants per slice) with a Nikon digital camera. The osteoclast-resorbing pits were stained deep blue. The pit areas were quantified using Scion image software (Scion Corporation, Frederick, MD, USA), and the data were expressed as the pit area/the total area.

### 4.6. Real-Time Quantitative Polymerase Chain Reaction

Total RNA was extracted from the co-cultured MLO-Y4 cells and RAW264.7 cells using Trizol reagent(Invitrogen, Carlsbad, CA, USA). cDNA was synthesized using the reverse transcription of 2 µg RNA in a 20-µL reaction mixture using SuperScript III (Invitrogen, Carlsbad, CA, USA). Real-time quantitative PCR was performed using specific primers for the above proteins and SYBR Green I Master Mix (Invitrogen) in an ABI 7900 cycler. Each experiment was repeated at least three times. The primers used were as follows: M-CSF (forward, 5′-ACCGAGAGGCTCCAGGAACT-3′; reverse, 5′-GTTCGGACACAGGCCTTGTT-3′); RANKL (forward, 5′-GCACACCTCACCATCAATGC-3′; reverse, 5′-AGCCTCGATCGTGGTACCAA-3′); OPG (forward, 5′-GACAACGTGTGTTCCGGAAA-3′; reverse, 5′-GCCTCTTCACACAGGGTGACA-3′); and β-actin (forward, 5′-ATCTGGCACCACACCTTC-3′; reverse, 5′-AGCCAGGTCCAGACGCA-3′).

### 4.7. ELISA for RANKL and M-CSF

The levels of soluble RANKL, OPG and M-CSF in the co-culture supernatant were measured on day 2, day 4, day 6, and day 8 using an ELISA kit (Abcam). The values were normalized to the total protein content.

### 4.8. Western Blot

Total protein was prepared using a whole-cell lysis assay kit (KeyGEN BioTECH, Nanjing, China). The samples were subjected to 5%–10% SDS-polyacrylamide gel electrophoresis (SDS-PAGE) and then transferred to a nitrocellulose membrane. After blocking with milk, the target proteins were detected with specific antibodies and then exposed to HRP-conjugated secondary antibodies. The blots were detected using electrochemiluminescence (ECL, Merk Millipore, Billerica, MA, USA).

### 4.9. Verification Tests

To test the hypothesis that the interaction between heparin and OPG impaired the activity of OPG, resulting in increased osteoclast formation, several verification tests were performed.

#### 4.9.1. The “Saturation Phenomenon” Test

The co-cultures were supplemented with heparin at very high doses (from 100 to 1000 IU/mL). Eight days later, the osteoclasts were counted.

#### 4.9.2. The Effects of Heparin on the Monoculture

RAW264.7 cells were monocultured and induced with 50 ng/mL RANKL, and various concentrations of heparin were added with or without supplementation with 10 ng/mL OPG. Six days later, the osteoclasts were counted.

#### 4.9.3. The Effects of Exogenous OPG on Heparin Activity

Various concentrations of exogenous OPG were added to the co-cultured cells with or without the addition of 100 IU/mL heparin. The osteoclasts were stained and counted on day 8.

#### 4.9.4. Osteoclast Formation Supported by Conditioned Media of MLO-Y4 Cells

To understand the effect of heparin, several types of MLO-Y4 conditioned media were prepared. RAW264.7 cells were then cultured in these conditioned media for eight days, and the osteoclasts were counted.

(1) MLO-Y4 cells were pre-treated with different levels of heparin for six hours and then cultured in fresh medium. After two hours (to test the early response) or 24 h (to test the late response), the medium was collected and supplemented with 5 ng/mL RANKL (the optimal minimum concentration to induce osteoclast formation) and reserved as heparin pre-treated MLO-Y4 conditioned medium (2h Hpt-Y4 and 24h Hpt-Y4; Figure 6A);
(2) The MLO-Y4 cells were normally cultured for 48 h, and the supernatant was collected. Through incubating this supernatant with various concentrations of heparin and adding with 5 ng/mL RANKL, heparin-incubated MLO-Y4 conditioned media (Hin-Y4) were obtained (Figure 6A). The RAW264.7 cells were cultured in Hin-Y4 conditioned medium for eight days, and osteoclast formation was assessed. Osteoclasts induced by empty MLO-Y4 supernatant with only the addition of 5 ng/mL RANKL served as the control;
(3) To determine the role of OPG in heparin’s effect on osteoclast formation, we set up three major groups, and the conditioned medium was prepared as follows (Figure 7A):
  • NONE groups: adding recombinant RANKL to empty Y4 supernatant (final concentration is 5 ng/mL) and then incubating with or without 100 IU/mL heparin;
  • Anti-OPG groups: adding recombinant RANKL to empty Y4 supernatant (final concentration was 5 ng/mL), then incubating with OPG neutralizing antibody (final concentration was 5 µg/mL), and finally incubating with or without 100 IU/mL heparin;
  • Exogenous OPG groups: replenishing exogenous OPG (final concentration was 20 ng/mL) in the conditioned medium of the anti-OPG groups.

### 4.10. Statistical Analysis

The data were analyzed using one-way ANOVA with Tukey *post hoc* analysis. Each experiment was repeated at least three times, and the results are expressed as the means with standard deviations. Analyses were performed with Statistical Analysis for Social Sciences software (SPSS v. 16.0, Armonk, NY, USA). The significance level was α = 0.05.
